# The Most Recently Discovered Carbonic Anhydrase, CA XV, Is Expressed in the Thick Ascending Limb of Henle and in the Collecting Ducts of Mouse Kidney

**DOI:** 10.1371/journal.pone.0009624

**Published:** 2010-03-10

**Authors:** Sina Saari, Mika Hilvo, Peiwen Pan, Gerolf Gros, Nina Hanke, Abdul Waheed, William S. Sly, Seppo Parkkila

**Affiliations:** 1 Institute of Medical Technology, University of Tampere, Tampere, Finland; 2 School of Medicine, University of Tampere, Tampere, Finland; 3 Center for Laboratory Medicine, Tampere University Hospital, Tampere, Finland; 4 VTT Technical Research Centre of Finland, Espoo, Finland; 5 Zentrum Physiologie, Medizinische Hochschule Hannover, Hannover, Germany; 6 Edward A. Doisy Department of Biochemistry and Molecular Biology, Saint Louis University School of Medicine, St. Louis, Missouri, United States of America; Sun Yat-Sen University, China

## Abstract

**Background:**

Carbonic anhydrases (CAs) are key enzymes for physiological pH regulation, including the process of urine acidification. Previous studies have identified seven cytosolic or membrane-bound CA isozymes in the kidney. Recently, we showed by *in situ* hybridization that the mRNA for the most novel CA isozyme, CA XV, is present in the renal cortex. CA XV is a unique isozyme among mammalian CAs, because it has become a pseudogene in primates even though expressed in several other species.

**Methodology/Principal Findings:**

In the present study, we raised a polyclonal antibody against recombinant mouse CA XV that was produced in a baculovirus/insect cell expression system, and the antibody was used for immunohistochemical analysis in different mouse tissues. Positive immunoreactions were found only in the kidney, where the enzyme showed a very limited distribution pattern. Parallel immunostaining experiments with several other anti-CA sera indicated that CA XV is mainly expressed in the thick ascending limb of Henle and collecting ducts, and the reactions were most prominent in the cortex and outer medulla.

**Conclusion/Significance:**

Although other studies have proposed a role for CA XV in cell proliferation, its tightly limited distribution may point to a specialized function in the regulation of acid-base homeostasis.

## Introduction

Carbonic anhydrases (CAs) are zinc metalloenzymes that function as regulators of systemic acid-base homeostasis by catalyzing the interconversion of carbon dioxide and bicarbonate. Sixteen members of the α-CA gene family have been found, from which 13 possess catalytic activity [1]. CAs are distributed in different tissues to participate in a variety of physiological processes, including urine acidification. In the kidney, at least seven isozymes (CA II, IV, IX, XII, XIII, XIV, and XV) have been identified [1], [2], [3], [4], [5], [6], [7], [8], [9], [10], [11]. Most of these isozymes are associated with the plasma membrane, except for cytosolic CA II and XIII [2], [3], [4], [9], [11]. Nonetheless, about 95% of all CA activity in the kidney is cytosolic and probably accounts for the high activity enzyme, CA II. Most of the remaining activity has been attributed to CA IV, CA XII, and CA XIV [7], [10], [12], [13]. Although the expression of different isozymes varies along the nephrons of different species, CA II seems to be the most widely distributed isozyme, being present in the intercalated cells of the collecting ducts as well as in the proximal tubules and the loop of Henle [14]. Both CA II and CA IV have been reported to associate with bicarbonate transporters [15]. Of the five membrane-bound CAs, CA IV is the most extensively expressed and has been found in the thick ascending limb and S2 segments of the proximal tubules of the rat kidney [5], and also in the intercalated cells of the rabbit collecting duct [16]. CA IV has been located predominantly at the luminal membranes, and some expression has also been reported at the basolateral membranes [5], [17]. The luminal CA activity was long thought to be solely attributable to CA IV until the two novel CAs, CA XIV and CA XV, were isolated and characterized. CA XII was originally identified as a tumor-associated isozyme [18], [19], but it was soon also demonstrated at the basolateral membranes in both S1 and S2 segments of the proximal tubules as well as in the cortical and outer medullary collecting ducts of the rat and mouse kidney [7]. In addition, it was found in the thick ascending limbs and distal convoluted tubules of the human kidney [8]. CA XII is most closely related to the other transmembrane isozyme, CA XIV, and their CO_2_ hydration activities are in the same range [20]. However, their subcellular locations are different: CA XII is confined to the basolateral membranes, whereas CA XIV is predominantly located at the luminal membranes. CA XIV is expressed in the thin descending limbs of Henle and S1 segments of the proximal tubules, and it may account for a considerable fraction of the luminal activity previously attributed to CA IV [10]. Besides CA II, CA XIII is another cytosolic isoform located in the kidney and has been found in the collecting ducts and renal corpuscle [9]. Although the low activity enzyme, CA III, may not be present in the normal kidney, it has been detected in mice and patients with proximal tubule dysfunction [21]. Transmembrane CA IX is weakly expressed in the rodent kidney [6], but its expression is highly induced in renal cell carcinoma [22], [23], [24].

CA XV, the most recently discovered CA isozyme, is most closely related to CA IV. Both isozymes have N-linked glycosylations and are attached to the membrane via a glycosylphosphatidylinositol (GPI)-anchor at the C-terminus of the polypeptide [1], [25]. CA XV is the first member of the α-CA gene family that is expressed in several species, but its gene has become a pseudogene in primates. This finding has to be considered in context with the presence of the high activity CA IV in humans and the low activity CA IV in rodents [26]. Thus, predominantly apical CA XV and CA XIV enzymes could functionally compensate the lower activity of CA IV in rodents. The presence of the high activity CA IV enzyme could explain why CA XV is no longer needed in primates.

Since our previous study [1] showed that, among other species, CA XV is also active in rodents, we examined the distribution of *Car15* mRNA in mouse tissues with RT-PCR and *in situ* hybridization. RT-PCR of *Car15* mRNA showed a strong band for the kidney and weaker bands for the brain, testis, and 7-day and 17-day embryos. *In situ* hybridization showed the most abundant expression in the renal cortex. Recombinant mouse CA XV was expressed in a baculovirus/insect cell expression system [27], and a polyclonal antibody was raised against the purified recombinant protein. In the present study, the new antibody was used for the immunohistochemical localization of CA XV in mouse kidney. The distribution of CA XV was compared with CA II, IV, XII and XIV. Immunostainings were also performed on *Car4^−/−^* and *Car14^−/−^* kidney samples, and the reactions were compared to those observed in the wild type kidneys. We also performed quantitative real time polymerase chain reactions (qRT-PCR) to see if there were any changes in *Car15* mRNA expression between the *Car4^−/−^*, *Car14^−/−^* and wild type kidneys.

## Materials and Methods

### Antibodies

A polyclonal rabbit antibody against the recombinant mouse CA XV [27] was raised in a rabbit by Innovagen AB (Lund, Sweden) and used to detect CA XV in tissue sections. For comparison, other anti-CA sera were also used, including rabbit anti-mouse CA II [9], rabbit anti-mouse CA IV [28], rabbit anti-mouse CA XII [7], and rabbit anti-mouse CA XIV [10], [29]. A rabbit antibody against Tamm-Horsfall glycoprotein (THP) (Santa Cruz Biotechnology, Santa Cruz, CA, USA) was used to identify the thick ascending limb of Henle in kidney sections.

### Western Blotting

The rabbit anti-mouse CA XV antibody was first tested by Western blotting. Recombinant mouse CA XV produced in Sf9 insect cells was subjected to standard sodium dodecyl sulfate-polyacrylamide gel electrophoresis (SDS-PAGE), and proteins were transferred to polyvinylidene difluoride (PVDF) membranes (Bio-Rad Laboratories, Richmond, CA). After transblotting, the membranes were treated for 25 minutes with Tris-buffered saline (TBS)+0.3% Tween 20 (TBST) containing 10% cow colostral whey (Hi-Col, Oulu, Finland), and then incubated for one hour with polyclonal anti-CA XV or pre-immune serum, each diluted 1∶500 with 1% bovine serum albumin (BSA) in phosphate-buffered saline (PBS). Membranes were washed five times for 5 minutes in TBST and then incubated for one hour with horseradish peroxidase-linked donkey anti-rabbit IgG (Amersham Sciences, Little Chalfont, UK) diluted 1∶25,000 in BSA-PBS. After washing the membranes four times for 5 minutes in TBST, the antibody binding was visualized by enhanced chemiluminescence (ECL; Amersham Sciences, Little Chalfont, UK).

### Immunohistochemistry

Mouse tissue sections obtained from several NMRI mice (at the Animal Care Center of the University of Tampere, Finland) were cut into 3 µm-thick sections and dried onto Superfrost Plus™ microscope slides. For comparison, tissue specimens were also obtained from three *Car4^−/−^* [30], four *Car14^−/−^* [30], and four wild type C57BL6 mice. The tissues included the kidney, liver, stomach, small intestine, colon, spleen, pancreas, heart, lung, and brain. Mouse tissue collection was conducted according to the provisions of the European convention for the protection of vertebrate animals used for experimental and other scientific purposes (Strasbourg, France). According to the national guidelines, no permission was required by authorities to collect tissue specimens from sacrificed mice.

The immunostaining method included the following steps: (1) deparaffinization of the sections using xylene and ethanol series; (2) treatment with 3% H_2_O_2_ in methanol for 5 min; (3) rinsing in TBS; (4) blocking with Rodent Block M™ (Biocare Medical, Concord, CA) for 30 min; (5) washing 3 times for 5 min with TBS; (6) incubation with polyclonal anti-CAs or anti-THP for 1 h (all the other antibodies except for anti-CA IV) or 2 h (anti-CA IV); (7) washing 3 times for 5 min with TBS; (8) incubation with a mixture of Rabbit on Rodent HRP-Polymer™ and blocking reagent XM Factor™ (20 µl XM Factor™ in 1 ml HRP-Polymer) (Biocare Medical, Concord, CA) for 30 min; (9) washing 3 times for 5 min with TBS; (10) incubation in DAB (3,3′-diaminobenzidine tetrahydrochloride) solution (Zymed, Carlsbad, CA) for 5 min; (11) rinsing in ddH_2_O; (12) counterstaining with hematoxylin (13); and rinsing again with ddH_2_O. The sections were mounted in Entellan Neu™ (Merck; Darmstadt, Germany) and photographed with a Zeiss Axioskop 40 microscope (Carl Zeiss; Göttingen, Germany).

### Quantitative Real Time PCR

mRNA was isolated from 15 mouse kidneys obtained from five *Car4^−/−^*, five *Car14^−/−^*, and five wild type C57BL6 mice. The kidneys were homogenized (Heidolph Silent Crusher S, Colonial Scientific, VA, USA), and the RNA was isolated using an RNeasy Mini Kit™ (Qiagen Sciences, MD, USA). The RNA concentration and purity were determined by NanoDrop™ (ThermoScientific, DE, USA). One microgram of total RNA was used as the template in reverse transcription (RT) and polymerase chain reaction (PCR). The RT reaction was performed at 37°C for 2 h followed by denaturation at 85°C for five minutes. The PCR cycling was performed at 50°C for 2 minutes, 95°C for 10 minutes, followed by 40 cycles of denaturation at 95°C for 15 seconds and annealing and elongation at 58°C for one minute. Dissociation was conducted at 95°C for 15 seconds. Primers for mouse glyceraldehyde-6-phosphate dehydrogenase (mGAPDH) were used to monitor the quality and quantity of cDNA.

Quantitative real time PCR was conducted using SYBR Green measured with ABI7000 (AME bioscience, Norway). Primers for murine *Car15* were the same as described in a previous study [1]. Measurements were performed in duplicate with 0.5 µl cDNA. Transcripts for the housekeeping gene *GAPDH* were also measured, and the values of *Car15* were expressed relative to the *GAPDH* expression.

## Results

### Localization of CA XV

**Figure 1:**
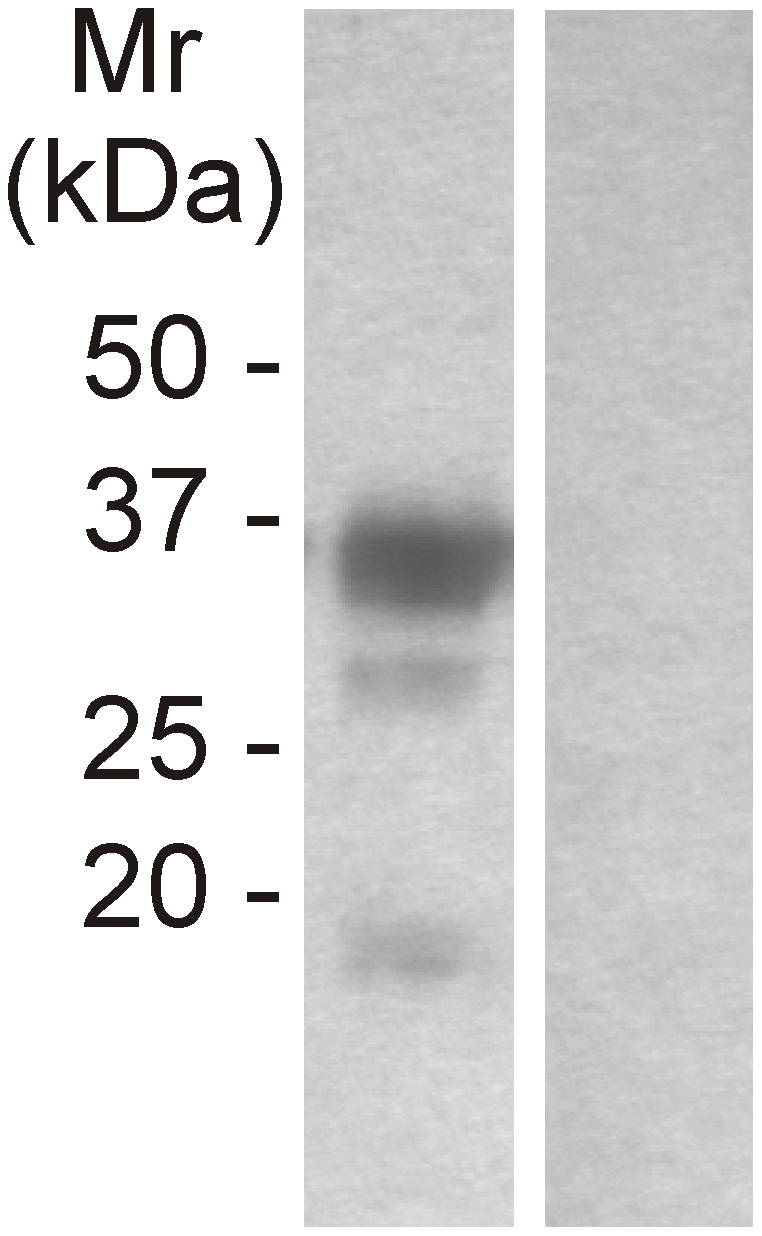
Western blot of recombinant mouse CA XV identified with the new anti-CA XV antibody. The antibody identifies a 34- to 36-kDa polypeptide and a smaller 31-kDa polypeptide that is suggested to be a non-glycosylated form of CA XV (left). Pre-immune serum showed no reaction (right).

The novel rabbit anti-mouse CA XV serum was first tested by Western blotting. The antiserum identified a strong 34- to 36-kDa polypeptide band and a smaller 31-kDa band on the Western blot of the purified recombinant mouse CA XV (Fig. 1). The result was in line with the earlier study where the 34- to 36-kDa polypeptide represented mature CA XV, while the 31-kDa polypeptide was a non-glycosylated form of CA XV [1]. Pre-immune serum showed no reaction.

**Figure 2:**
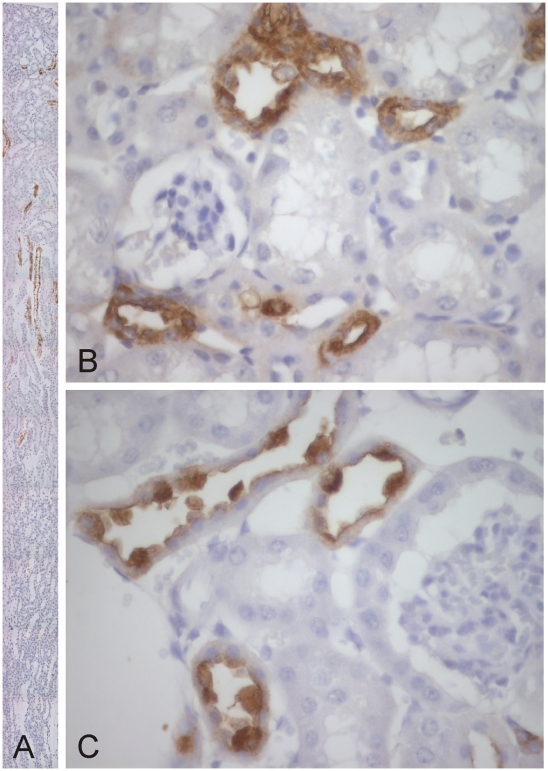
Immunohistochemical staining of CA XV in the mouse kidney. A sectional view of mouse kidney shows that CA XV is expressed in the cortex, and a weaker reaction is seen in the collecting ducts of the outer medulla (A). Intense staining is seen in the thick ascending limbs (B) and collecting ducts of the cortex (C). Original magnifications x100 (A), x630 (B,C).

The localization of CA XV was studied by immunohistochemistry in mouse tissue samples. Kidney was the only tissue showing positive immunoreaction for CA XV, and the strongest signal was observed in the cortex and occasional staining the outer medulla (Fig. 2A). In these regions, CA XV seemed to be predominantly expressed in the thick ascending limb of Henle and collecting ducts (Fig. 2B, C). The weak staining observed deeper in the medulla was located in the collecting ducts.

**Figure 3:**
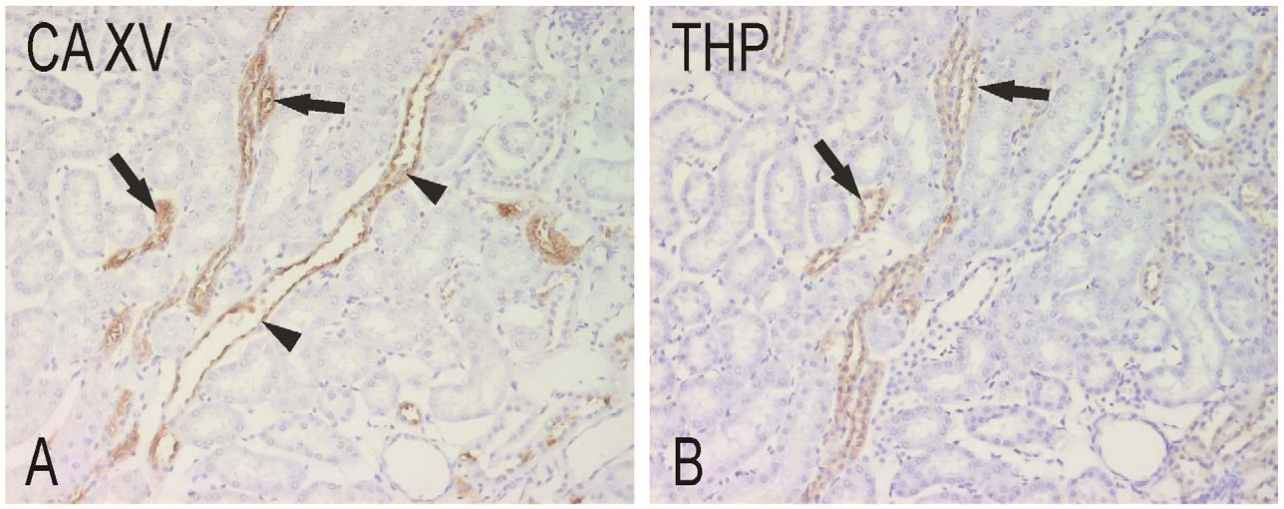
Confirmation of CA XV immunostaining in the thick ascending limb of Henle. Tamm-Horsfall glycoprotein (THP) antibody was used as a marker (B). CA XV (A) clearly labels the thick ascending limbs (arrows) and collecting ducts (arrowheads). Original magnifications x400.

To confirm the location of the positive signal, an antibody against Tamm-Horsfall glycoprotein was used as a marker of thick ascending limbs [10]. Figure 3(A, B) shows that the Tamm-Horsfall glycoprotein-positive tubules were also positively identified in the CA XV immunostaining. In general, the marker gave a fainter reaction in comparison to CA XV.

**Figure 4:**
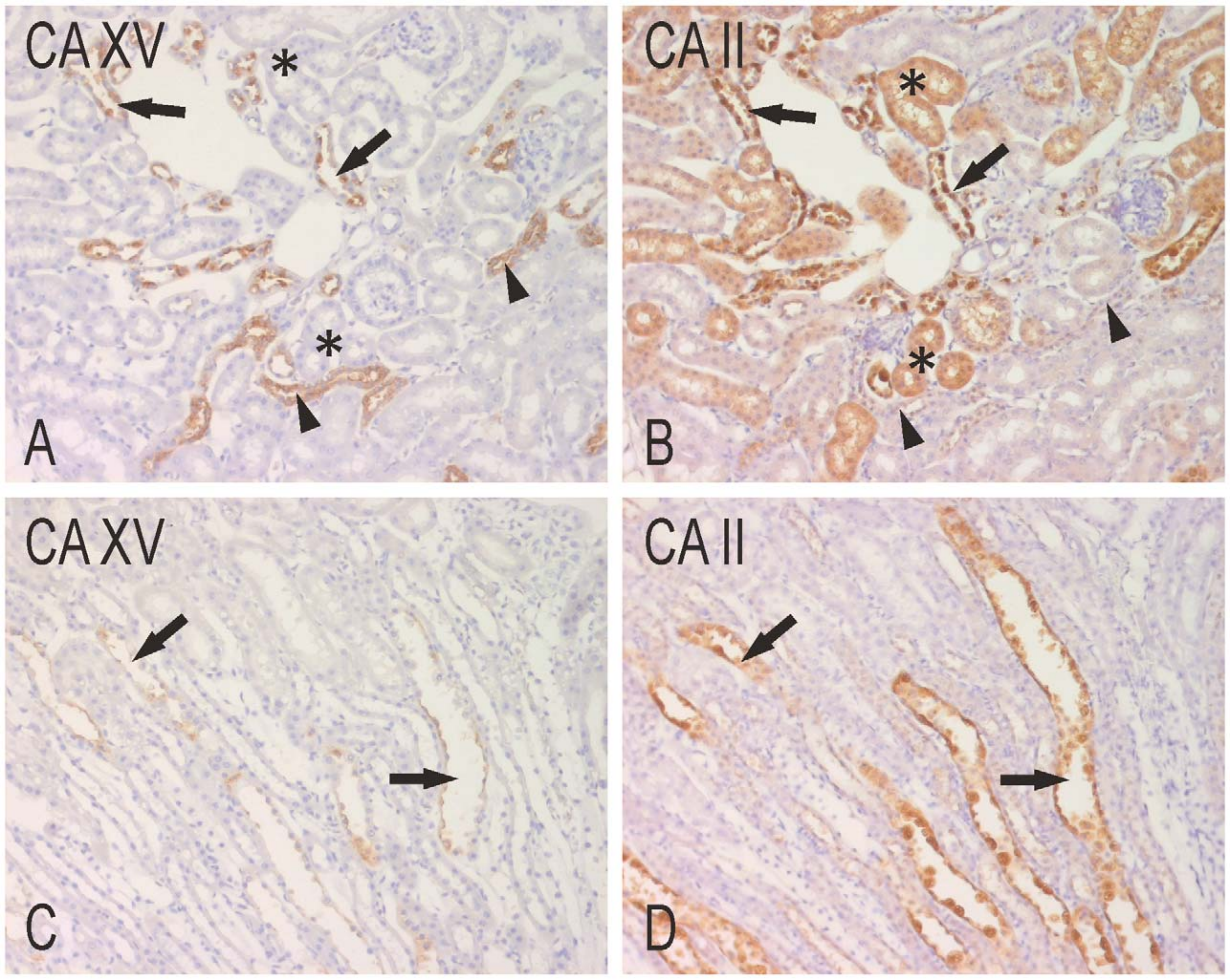
Immunohistochemical staining of CA XV and CA II in parallel sections of mouse kidney. In the cortex, the expression of CA XV (A) is more limited compared to CA II (B) that is mainly expressed in the collecting ducts (arrows) and proximal tubules (asterisks). Arrowheads show the thick ascending limbs of Henle, which are positively stained for CA XV. In the medulla, only a very faint reaction is seen for CA XV (C) in the collecting ducts (arrows), while the staining for CA II (D) is more intense. Original magnifications x400.

We also performed immunostainings for CA XV and the other four renal CAs, including CA II, IV, XII, and XIV, in parallel tissue sections. The results showed that the distribution pattern of CA XV was unique among all the other isozymes. CA II-staining was more intense and widely spread than CA XV, as expected, involving both the proximal convoluted tubules and collecting ducts. CA XV was co-expressed in the cortical collecting ducts, whereas the reactions for CA XV were much weaker in the medulla (Fig. 4). The CA XV-positive structures identified as the thick ascending limbs of Henle remained negative for CA II.

**Figure 5:**
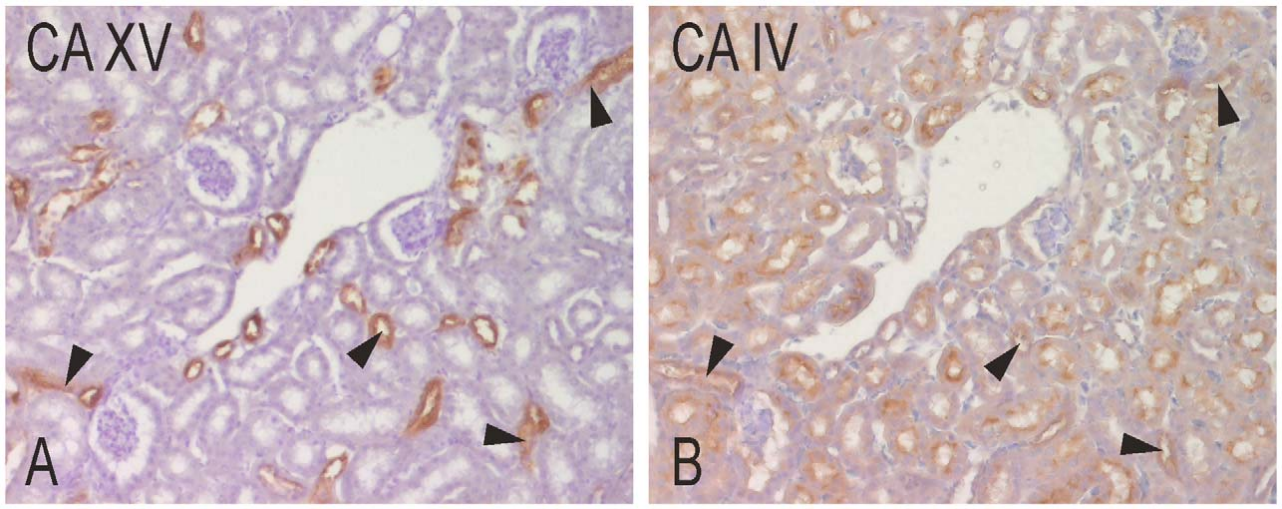
Immunohistochemical staining of CA XV and CA IV in mouse kidney. The staining for CA XV (A) is more intense and restricted compared to CA IV (B). CA IV shows a reaction in the thick ascending limbs (arrowheads) like CA XV, and is also present in the proximal tubules, which are not stained for CA XV. Original magnifications ×400.

According to the phylogenetic studies, CA IV is the most closely related isozyme to CA XV, and they both showed expression in the thick ascending limbs (Fig. 5). The proximal convoluted tubules also showed positive reaction for CA IV, as reported earlier [5]. More background signal was seen in the CA IV-staining due to a longer incubation time with the primary antibody.

**Figure 6:**
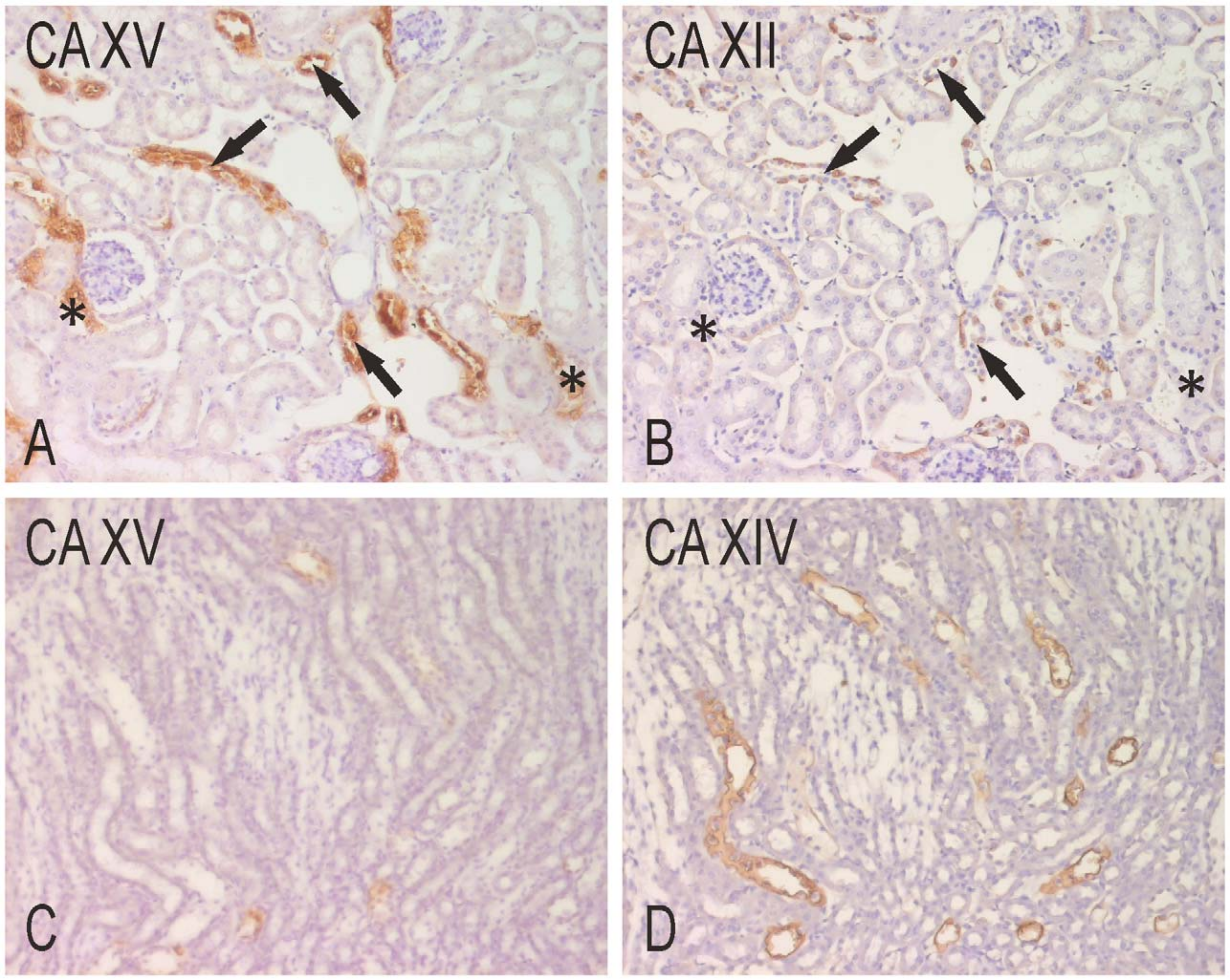
Immunohistochemical staining of CA XV, CA XII and CA XIV in mouse kidney. CA XV (A) and CA XII (B) label the same cortical collecting ducts (arrows), even though the reaction for CA XV is notably more extensive and intense. CA XV is also present in the thick ascending limbs of Henle (asterisks), which are negative for CA XII. In the medulla, CA XIV (D) is expressed in the upper portion of the thin descending limbs, as described previously [10]. These segments are negative for CA XV (C). Original magnifications ×400.

CA XII has been localized to the mouse proximal convoluted tubules and more strongly to the collecting ducts [7]. In our study, CA XII showed very weak reactions in the proximal tubules and strong signal in the collecting ducts; the collecting ducts also showed strong reactions for CA XV (Fig. 6). CA XIV is predominantly expressed in the thin descending limbs of Henle and proximal convoluted tubules [10]. Therefore, CA XIV-positive structures were distinct from those of CA XV (Fig. 6).

**Figure 7:**
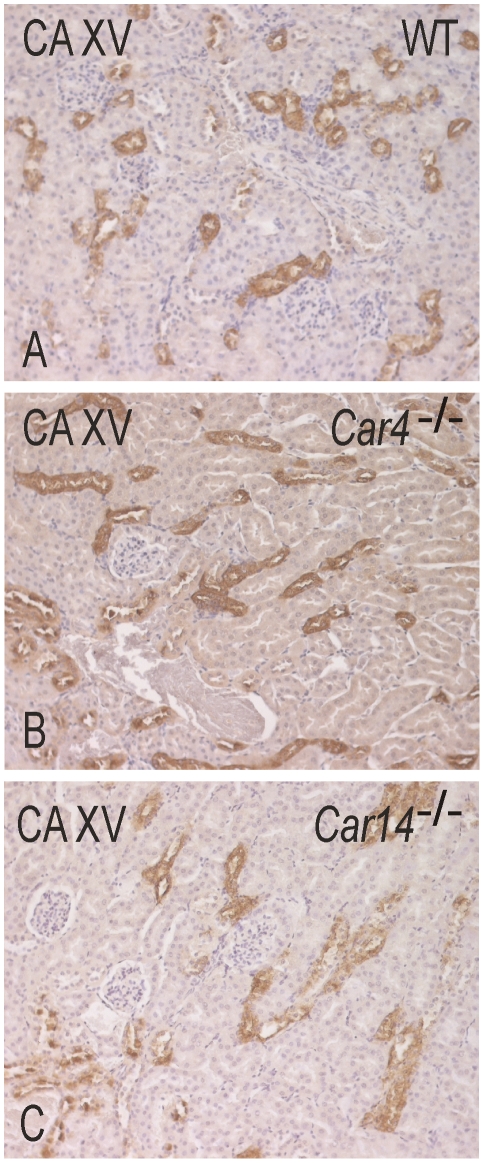
Immunohistochemical staining of CA XV in wild-type, *Car4^−/−^* and *Car14^−/−^* mouse kidneys. In addition to the strong immunoreactions observed in all the cases in the collecting ducts and thick ascending limbs, weak positivity is also observed in the convoluted tubules of *Car4^−/−^* kidney. Original magnifications ×400.

CA XV was also localized in the kidney specimens obtained from *Car4^−/−^* and *Car14^−/−^* mice to see if the absence of either CA IV or XIV caused any changes in the amount of CA XV immunoreactivity. The kidney was the only CA XV-positive tissue in the knockout mice like in the wild type mice. One kidney specimen obtained from a *Car4^−/−^* mouse showed a more widespread immunostaining for CA XV including moderate reactivity in the proximal convoluted tubules (Fig. 7). In the other specimens, we observed no significant change in the expression levels or distribution patterns.

### Quantitative Real-Time PCR

**Figure 8:**
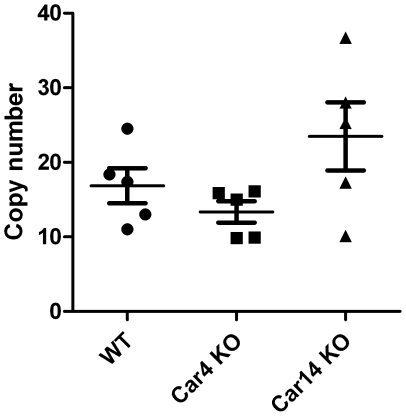
Quantitative real-time PCR analysis of *Car15* mRNA levels in the kidney specimens of wild type and *Car4^−/−^* and *Car14^−/−^* knockout mice. Three of five kidney samples from *Car14^−/−^* knockout (KO) mice showed slightly increased expression of *Car15* mRNA in comparison to the wild type (WT) and *Car4^−/−^* kidneys, but the difference between the groups showed no statistical significance.

Because immunohistochemical staining showed higher reactivity in one *Car4^−/−^* kidney than in wild type or *Car14^−/−^* kidneys, we performed quantitative real time PCR to examine *Car15* mRNA levels in the kidney specimens of wild type, *Car4^−/−^* and *Car14^−/−^* mice. The results were expressed relative to the amounts of transcripts of the housekeeping gene *GAPDH*. There were no significant changes in the mRNA levels (Fig. 8). Unexpectedly, three of five kidney samples from *Car14^−/−^* mice showed increased expression of *Car15* mRNA in comparison to the wild type and *Car4^−/−^* kidneys, but the difference showed no statistical significance according to the chi-square (χ^2^) test.

## Discussion

Urine acidification is regulated by two major processes: proton secretion and bicarbonate reabsorption. Among different CA isozymes, CA II is probably the main contributor to the production of protons in different sections of the nephron and collecting ducts. There is also general agreement about the importance of the proximal tubule in bicarbonate reabsorption [31]. However, other downstream segments participate in this process as well [32]. In fact, the loop of Henle, under normal conditions, is able to reabsorb about 15% of the filtered bicarbonate [33]. Membrane-associated CA activity is apparently involved in this process. Even though the minority of all CA activity in the kidney is membrane-associated [12], [13], five isozymes have been discovered to be responsible for it [1], [5], [6], [7], [10]. All membrane-bound CA activity was long attributed to CA IV. In rodents, it is expressed on the apical membranes of the thick ascending limbs of Henle and proximal tubules and less so on the basolateral side of the epithelial cells [5]. CA XII was originally identified as a tumor-associated isozyme, which was localized to the basolateral membranes of the proximal tubules and collecting ducts in rodents [7]. CA XIV is distributed on both the basolateral and apical membranes of the thin descending limbs of Henle and proximal tubules, and it has been suggested to play a key role in bicarbonate reabsorption along with CA IV [10]. In a recent study, the results from RT-PCR and *in situ* hybridization suggested that CA XV, the most novel member of the α-CA gene family, might be expressed in the renal cortex [1]. Our results showed positive signal for CA XV only in the kidney, and, therefore, the distribution of CA XV appears to be very limited as compared to all other mammalian CA isozymes. In the kidney, most CA XV was located in the cortex, and weaker immunoreactions were seen in the medulla, which was consistent with the earlier *in situ* hybridization results [1]. The expression was shown specifically in the thick ascending limbs and collecting ducts of the cortex and outer medulla.

CA IV is expressed in several different tissues. In addition to the kidney, it has been found, for example, in the heart, intestine, lung, and gallbladder [25], [34], [35], [36], [37]. According to our previous phylogenetic studies, CA XV is most closely related to CA IV, and these two isozymes share several similar structural properties [1]. The staining showed expression partially in the same structures along the nephron, and, unlike the other membrane-bound isozymes, both CA IV and CA XV are attached to the membrane by a GPI-anchor. CA XV also contains N-linked glycosylations and stabilizing disulfide linkages, like CA IV [1]. The activity of murine CA IV is only 10-20% of that of human CA IV [26], which has raised the question of whether the two isozymes, CA XIV and XV, could compensate for this loss of activity in the rodent kidney. Unlike CA XV, which has become a pseudogene in humans and chimpanzees, CA XIV is present in several human tissues, including the heart, liver, skeletal muscle, and brain [29], [37], [38], [39], but the human enzyme has significantly lower activity than the mouse enzyme [40]. Although CA XIV has been partially localized to the same nephron segments where CA IV is also produced [5], [10], CA XIV showed no overlapping with CA XV in its distribution.

In rodent kidney, recent studies have suggested that CA XIV may have a more important role than CA IV in bicarbonate reabsorption in proximal tubular cells, because it is expressed in both S1 and S2 segments, while CA IV is mainly present in S2 [10]. An interesting discovery was the unexpected halo-tolerance of CA XIV, a physiological property that allows the isozyme to stay active at a lower pH than CA IV [41]. The functional significance of CA XIV may not only be to compensate for the lower activity of murine CA IV as expected but also to adapt to a more challenging environment.

In addition to proximal tubules, CA IV is expressed in the thick ascending limbs of Henle [5] together with CA XV. Based on the similar localization, one could argue that these two enzymes can share the same role in bicarbonate reabsorption within this segment. However, it was recently proposed that CA XV might participate in the control of cell proliferation during acidosis and potassium depletion in the collecting ducts [42]. CA XV showed a different amplitude and time course of over-expression compared to other acid-base transport proteins, supporting the hypothesis that CA XV unlikely contributes to acid-base homeostasis in the collecting ducts.

In the present study, we also evaluated the possible compensatory changes in *Car15* gene expression when either CA IV or CA XIV was absent. In one specimen of *Car4^−/−^* kidney, we observed some reactivity for the CA XV antibody, especially in proximal tubules, that was not evident in the wild type kidneys. However, when measured with quantitative real time PCR, there seemed to be no significant change in the expression of *Car15* mRNA in *Car4^−/−^* kidney as compared to the wild type mice. This result indicated that the transcription of *Car15* gene is not increased in CA IV deficient mice, and the positive reaction of the proximal tubules may be due to an artifact caused by nonspecific binding of the antibody. By immunohistochemistry, no change was detected in the distribution of CA XV in *Car14^−/−^* kidneys compared to the wild type specimens, but quantitative real time PCR unexpectedly showed some mild up-regulation of *Car15* gene expression in three of five *Car14^−/−^* kidneys. Even though the difference did not reach statistical significance, this slight tendency may point to some compensatory changes in occasional cases, depending on the physiological needs of the mice. Though the role of CA XV as a compensating isozyme for CA IV and CA XIV may not be as significant as presumed, it is not entirely excluded.

A study with knock-out mice showed that both CA IV and CA XIV contribute to buffering of the extracellular space in the brain [30]. The lack of both CA IV and CA XIV in double knock-out mice increased the amplitude of the alkaline transient, causing a delay in the alkaline shift. Neither the CA IV nor the CA XIV transcript levels were up-regulated when the other was absent, yet there was no significant change in pH-regulation in single knock-out mice compared to wild-type. These results suggested that the normal expression level of either CA IV or CA XIV is enough to compensate for the other isozyme. Both RT-PCR and *in situ* hybridization showed a positive signal for *Car15* mRNA in the brain [1], and, therefore, CA XV emerged as another candidate protein to contribute to extracellular buffering. However, we observed no immunohistochemical signal for CA XV in the normal or knock-out brain tissue (data not shown), suggesting that the protein is not produced in the brain or that the level of expression was below the detection limit of our staining protocol. Nevertheless, the membrane-associated isozymes may significantly contribute to the compensatory mechanisms in some cell types.
